# Inflammatory markers in acute myocardial infarction and the correlation with the severity of coronary heart disease

**DOI:** 10.1080/07853890.2021.1916070

**Published:** 2021-06-28

**Authors:** Nicoleta Oprescu, Miruna Mihaela Micheu, Alexandru Scafa-Udriste, Nicoleta-Monica Popa-Fotea, Maria Dorobantu

**Affiliations:** a Department of Cardiology, Clinical Emergency Hospital of Bucharest, Romania; b Cardio-thoracic Department, University of Medicine and Pharmacy “Carol Davila”, Bucharest, Romania

**Keywords:** Inflammatory markers, IL-1β, CRP, SDF-1α, acute myocardial infarction, coronary stenosis severity, Gensini score

## Abstract

**Introduction:**

The inflammatory hypothesis of atherosclerosis is appealing in acute coronary syndromes, but the dynamics and precise role are not established.

**Objectives:**

The study investigates the levels of C reactive protein (CRP), interleukin 1β (IL-1β) and stromal-derived factor 1α (SDF-1α) at the time of acute myocardial infarction (AMI) and at 1 and 6 months afterwards, compared with a control group.

**Results:**

In the acute phase of AMI, CRP and SDF-1α were significantly higher, while IL-1β showed lower levels compared with controls. CRP positively correlated with coronary stenosis severity (rho = 0.3, *p*=.05) and negatively related with left ventricle ejection fraction (LVEF) at 1 month (rho= −0.43, *p*=.05). IL-1β weakly correlated with the severity of coronary lesions (rho =0.29, *p*=.02) and strongly with LVEF (rho= −0.8, *p*=.05). SDF-1α, slightly correlated with LVEF at 1 month (rho = 0.22, *p*=.01) and with the severity of coronary atherosclerosis (rho= −0.41, *p*=.003).

**Conclusions:**

CRP, IL-1β and SDF-1α have important dynamic in the first 6 months after AMI and CRP and SDF-1α levels correlated with the severity of coronary lesions and LVEF at 1 month after the acute ischaemic event.

## Introduction

The role of inflammation in ischaemic heart disease has been outlined and identified several years ago [1,2]. Initial observational studies have shown that patients with acute coronary syndromes have elevated inflammatory markers (leukocytosis, neutrophilia, increased erythrocytes sedimentation rate, fibrinogen and C reactive protein [CRP]) [3]. CRP is an acute-phase protein and a well-known marker for detection, risk stratification and inflammation monitoring. CRP is a standard for highlighting the inflammatory process in acute coronary syndrome compared with the relatively recent inflammatory markers introduced into the clinic (interleukin [IL]-1β, IL-6 and stromal-derived factor 1α [SDF-α]). However, CRP is a parameter with low specificity, which shadows its diagnostic and prognostic value in acute myocardial infarction (AMI). In patients with AMI, CRP increases at 4–6 h after the onset of symptoms, reaches a peak concentration at 2–4 d, and its values ​​are approaching normal values after 7–10 d [4]. CRP concentrations are increased in obese patients, smokers and those with hormone replacement therapy and reduced by the use of statins, moderate alcohol consumption and exercise [5]. In primary prevention, CRP may play a role in predicting future cardiovascular events similar to total and HDL-cholesterol, and in secondary prevention, the risk of AMI recurrence, stroke and cardiovascular death [6]. For cardiovascular prevention, a concentration >3 mg/L is associated with a high risk, but a value ≥10 mg/L could be more appropriate for patients with acute coronary syndrome [7].

IL-1 is a pro-inflammatory mediator that induces the synthesis and expression of several secondary inflammatory mediators. Two distinct genes encode IL-1α and IL-1β proteins, both having the same cellular receptor (IL-1RI) [8]. IL-1β is the major circulating form of IL-1. Genetically engineered deletion of the IL-1β receptor has a protective response on animal model with AMI by ischaemia/reperfusion or severe non-reperfusion, by reducing infarcted areas, preventing ventricular dilation and systolic dysfunction of the left ventricle [8,9]. Administration of recombinant forms of IL-1 receptor antagonist, namely Anakinra, immediately after the onset of ischaemia or 24 h later results in a significant reduction of cardiomyocyte apoptosis and less ventricular dilation days after the permanent ligature of the coronary artery [10]. Anakinra, an antagonist recombinant IL-1 receptor was tested in a pilot study of feasibility on 10 subjects with AMI with ST-segment elevation [11] and subsequently on a group of follow-up of 30 patients [12], showing safety and tolerability, as well as a reduction in inflammation, decreasing the incidence of heart failure. In the Canakinumab Anti-Inflammatory Thrombosis Outcome Study (CANTOS) [13] on 10.061 subjects, it was demonstrated a reduction of the composite endpoint that included cardiovascular death, AMI and non-fatal stroke using Canakinumab, a monoclonal antibody anti-IL-1β. Although many studies have shown the release of IL-1α and IL-1β on AMI experimental models, the increase in IL-1 plasma levels in AMI patients was not systematically highlighted, some studies showing values ​​comparable to those of patients without coronary disease [14].

SDF-1α is a CXC chemokine with a number of distinct features. After AMI, SDF-1α is believed to contribute to the production of a favourable environment for stimulating stem cell adhesion to ischaemic tissue. For this, SDF-1α must be produced in the heart and released into circulation. SDF-1/CXCR4 system has been shown to have not only cardioprotective effects [15], but also angiogenic effects in several models of AMI [16]. Experimental data showed conflicting data on cardiac and plasma expression of SDF-1α, some authors reporting high values ^​​^ [17,18] and other insignificant changes [19].

## Objectives

Considering the central role of inflammation in the pathogenesis of atherothrombosis and atherosclerosis, this research area is in continuous development. Although several markers of inflammation are proposed, their role in the diagnosis and treatment of acute coronary syndromes is not yet well defined.

The objectives of this study are to determine the values ​​of inflammatory markers such as CRP, IL-1β and SDF-1α at the time of AMI with ST-segment elevation, as well as in dynamics at 1 and 6 months afterward and to compare its dynamics with a control group consisting of patients without significant coronary lesions, as well as the investigation of a possible correlation between the severity of coronary lesions, left ventricular ejection fraction and inflammatory status.

## Subjects

A total of 42 patients were enrolled in the study; 28 patients in the AMI group and 14 patients in the control arm. The inclusion criteria for the AMI group were as follows, age ≥18 years and documented spontaneous acute ST-elevation MI, diagnosed according to the universal MI criteria [20]. The control group included subjects with angina pectoris, older than 18 years old, with no history of MI or coronary revascularization (percutaneous coronary interventions or coronary artery bypass graft) and no significant atherosclerotic involvement of coronary arteries at angiography. From the study were excluded subjects with acute or chronic infections and autoimmune diseases. Written informed consent was obtained from all participants, while the study protocol was approved by the Ethics Committee of Emergency Clinical Hospital, Bucharest, Romania, in accordance with the Declaration of Helsinki. Traditional risk factors such as: age, sex, smoking, hypertension, dyslipidaemia, diabetes mellitus and body mass index were assessed for both groups.

All participants were investigated with a cardiac transthoracic echography; for the control subjects, it was realized at inclusion, and for AMI group patients at inclusion, before being discharged from the hospital after the AMI and during follow-up, at 1 and 6 months.

## Material and methods

### Sample collection and storage

Blood was harvested for inflammatory markers by peripheral venous puncture at 3 d after admission for AMI with ST-segment elevation, named T0, as well as at 1 and 6 months after the ischaemic event, named T1 and T6. Blood was harvested on EDTA tubes and worked within 30 min. Plasma was obtained by centrifugation, performed for 15 min at 1000 *g*. Subsequently, it was aliquoted and stored at < −20 °C. For the control group, blood was harvested at study enrolment.

### Inflammatory cytokines assay procedure

The method used for the quantitative determination of CRP, IL-1β and SDF-1α was the enzyme-linked immunosorbent assay (ELISA) method. The protocol for each of the three ILs was performed following the instructions of R&D Systems Human Kit designated for each cytokine in part (Human CRP Quantikine ELISA Kit, IL-1β Human IL-1β/IL-1β F2 Quantikine ELISA Kit and Human CXCL12/SDF-1α Quantikine ELISA Kit, R&D Systems Inc., Minneapolis, MN). The normal range of values for CRP was between 1.09 and 4.291 mg/L, with a mean of 1.769 mg/L, with the limit of detection 0.005–0.022 ng/mL (mean value 0.010 ng/mL) and no significant cross-reactivity or interference; the coefficient of variance for intra-assay precision was 3.8–4.3% and for inter-assay precision 6–7%. For IL-1β, the normal range of values was between 1 and 3.9 pg/mL, with the limit of detection 1 pg/mL and no significant cross-reactivity or interference; the coefficient of variance for intra-assay precision was in the range 2.8–8.5% and for inter-assay precision, 4.1–8.4%. For SDF-1α, the normal range of values was between 1330 and 2720 pg/mL (mean 1830 pg/mL), with the limit of detection 1.0–47 pg/mL (mean value 18 pg/mL) and no significant cross-reactivity or interference; the coefficient of variance for intra-assay precision was between 3.4% and 3.9% and for inter-assay precision, 8.2–13.4%

### Cardiac transthoracic echography

Echocardiographic parameters were measured with a 1.7/3.4 MHz transthoracic probe for 2D and with a 1.7/3.5 MHz transthoracic probe for 3D data acquisition from GE Healthcare Vivid E9. All data were analysed offline on EchoPac workstation. Standard sections were assessed end-diastolic and end-systolic volumes, as well as left ventricle systolic function appreciated with left ventricle ejection fraction (LVEF) by two-dimensional (2D) and three-dimensional (3D) echocardiography. The measurements were done by two independent operators trained in cardiac echography. Each investigator measured the parameters two times, separated in time, at least 2 weeks apart.

### Coronary angiography

All subjects included in the study underwent coronary angiography at inclusion. The interventional cardiologists were not informed by the existence of the two groups and the functional significance of the coronary plaques was reported with the use of Gensini score [21].

## Statistics

Average values were calculated for continuous variables and absolute frequencies for discrete parameters. For continuous variables, differences in the concentrations of CRP, IL-1ß and SDF-1α were examined by Mann–Whitney rank-sum or ANOVA assay, depending on the normal distribution of the variables and the differences between other variables (age, sex, total cholesterol, triglycerides, glycaemia, fibrinogen, leukocytes, neutrophils and lymphocytes percentage, ESR, LVEF and LV volumes) were calculated by the T-student test for independent variables. For the comparison of repeated measurements of ILs at T0, T1 and T6, since the distribution of values ​​did not follow normal distribution criteria, the non-parametric Friedman test was applied as recommended. Correlations were performed based on the Spearman test. Statistical values ​​of *p*≤.05 were considered statistically significant. The intra and inter-observer agreement for LVEF and LV volumes were assessed with intraclass correlation coefficient (ICC) and 95% confidence intervals (95% CIs). The level of agreement was appreciated as poor (<0.50), moderate (0.51– 0.75), good (0.76– 0.90) or excellent (>0.90).

## Results

The full characterization of the study population is shown in Table 1. It can be observed that the only statistically significant differences between the AMI and control groups are male gender, active smoking status, LVEF 2D and 3D, end-systolic and end-diastolic LV volumes, Gensini scores, CRP, IL-1β and SDF-1α. The Gensini score was 5.1 ± 0.9 points in controls, 15.1 ± 4.2 in AMI with single-vessel coronary artery disease (12 subjects), 22 ± 3.8 points in AMI with double-vessel coronary artery disease (14 subjects) and 29 ± 5.1 in those with multi-vessel coronary artery disease (2 subjects).

**Table 1.** Description of control and acute myocardial infarction groups.

Variables	AMI (*n* = 28)	Control (*n* = 14)	*p* Value
Age (years)	57.43 ± 12.8	57.9 ± 8.86	.09
Sex (men)	24 (88%)	8 (57%)	.006
Active smokers	16 (57%)	2 (14%)	.002
Diabetes mellitus type 2	17 (61%)	7 (50%)	.348
Arterial hypertension	22 (78.6%)	10 (78.5%)	.578
Total cholesterol (mg/dL)	207.93 ± 7.1	208.06 ± 21.8	.08
Triglycerides	171.95 ± 19.6	203.19 ± 43.3	.45
Glycaemia (mg/dL)	133.93 ± 10.2	115.5 ± 9.8	.21
Fibrinogen	610 ± 69	411 ± 44	.102
ESR	31.5 ± 3.7	11.3 ± 7.3	.097
Leucocytes number	9924.78 ± 378	9723.16 ± 808	.78
Neutrophils (%)	63.9 ± 2.1	57.11 ± 2.9	.21
Lymphocytes (%)	8.6 ± 0.56	9.7 ± 1.09	.37
Gensini score	21.2 ± 4.5	5.1 ± 0.9	.02
CRP (mg/L)	3.55 ± 0.4	2.2 ± 0.4	.043
IL-1β (pg/mL)	3.14 ± 0.74	12.6 ± 4.7	.05
SDF-1α (pg/mL)	2063.06 ± 183	1728.9 ± 143	.003
LVEF 2D (%)	41.2 ± 9.5	55.4 ± 3.78	.0001
LVEF 3D (%)	41.5 ± 8.5	56 ± 1.9	.0001
Drugs			
Calcium channels blockers	1 (3.67%)	1 (7%)	.46
Beta-blockers	13 (43%)	5 (36%)	.61
ACEI/ARBs	9 (32%)	4 (28%)	.78
Diuretics	1 (3.6%)	1 (7%)	.21
Statins	25 (89%)	7 (50%)	.07

Values ​​were expressed as number (percentage) or mean ± standard deviation.

ACEI: angiotensin conversion enzyme inhibitor; AMI: acute myocardial infarction; ARBs: angiotensin receptor blockers; CRP: C reactive protein; ESR: erythrocyte sedimentation rate; IL-1β: interleukin 1β; LVEF: left ventricle ejection fraction; SDF-1α: stromal derived factor 1α.

Aspirin was not included because all patients in the study received aspirin.

The plasmatic changes of the three pro-inflammatory markers measured with their statistical significance are found in Table 2.

**Table 2.** Evolution of inflammation in the acute myocardial infarction and control group.

		T0	T1	T6	*p* Value
CRP (mg/L)	Control	2.2 ± 0.4			.043
	AMI	3.55 ± 0.4	1.9 ± 0.33	1.78 ± 0.37	
IL-1β (pg/mL)	Control	1.26 ± 4.7			.05
	AMI	3.14 ± 0.74	8.2 ± 0.26	12.63 ± 4.44	
SDF-1α (pg/mL)	Control	1728.9 ± 143			.003
	AMI	2063 ± 183	2061 ± 169	2041 ± 189	

Values ​​were expressed as mean ± standard deviation.

AMI: acute myocardial infarction; CRP: C reactive protein; IL-1β: interleukin 1β; SDF-1α: stromal derived factor 1α.

**Figure 1.**
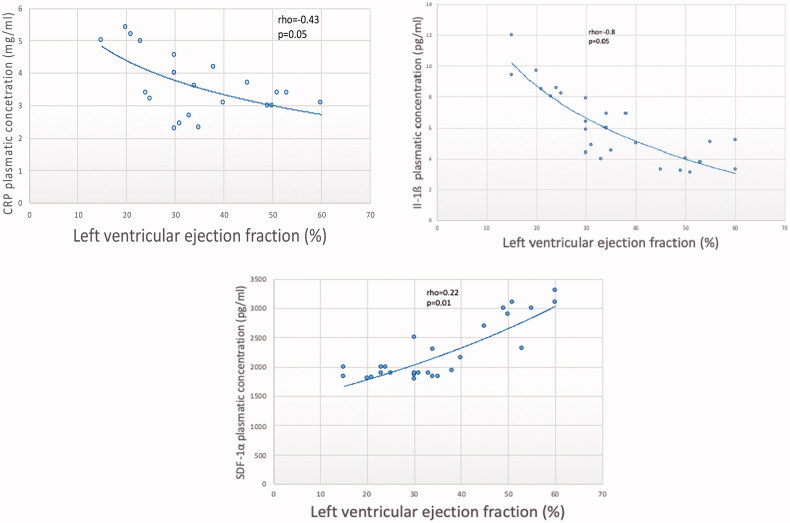
The correlation between inflammatory biomarkers in the acute phase of MI and two-dimensional left ventricular ejection fraction at 1 month. CRP: C reactive protein; IL-1β: interleukin 1β; SDF-1α: stromal-derived factor 1α

CRP is significantly higher in AMI patients compared to control (*p*=.043). Plasma concentrations of CRP decreased from baseline (3.55 ± 0.4 mg/L) at 1 (1.9 ± 0.33 mg/L) and 6 months (1.78 ± 0.37 mg/L). CRP values at 6 months in AMI patients were even lower (1.78 ± 0.37 mg/L) compared to controls (2.2 ± 0.4 mg/L), *p*<.05. It can be noticed an important dynamics of CRP values over the 6 months of follow-up, with a decrease statistically significant (*p*<.05) (Table 2). The CRP values at T0 weakly correlated with the extension and severity of coronary lesions (rho = 0.3, *p*=.05, CI: 0.1,0.67) and moderately with 2D and 3D LVEF at T1 (rho= −0.43, *p*=.05, CI: −0.69, −0.07; rho= −0.4, *p* = 0.05, CI: −0.67, −0.03) (Figure 1).

IL-1β was increased at T1 (8.2 ± 0.26 pg/mL) and T6 (12.63 ± 4.44 pg/mL) relative to baseline (3.14 ± 0.74 pg/mL). The increase in IL-1β values ​​from T0 to T1 and T6 is statistically significant (*p*<.05) (Table 2). IL-1βat T0 weakly correlated with the severity of coronary lesions (rho = 0.29, *p*=.02, CI: 0.09, 0.59), but strongly related with 2D and 3DLVEF at T1 (rho= −0.8, *p*=.05, CI: −0.9, −0.6; rho= −0.867, *p* = 0.015, CI: −0.93, −0.71) (Figure 1).

About SDF-1α dynamics, it was noted that a decrease at both at 1 month (2061 ± 169 pg/mL) and 6 months (2041 ± 189pg/mL) post-AMI. SDF-1α values never reach the control group level (1728.9 ± 143 pg/mL), remaining significantly elevated throughout the follow-up period. SDF-1α dynamics, although showing a decrease in concentration from baseline to T1 and T6, is not statistically significant (*p*=.102) (Table 2). SDF-1α at T0 correlated with the severity of coronary lesions (rho= −0.41, *p*=.003, CI: −0.67, −0.04), and with LVEF at 1 month (rho = 0.22, *p*=.01, CI: 0.06, 0.54) (Figure 1).

The values obtained for LVEF at T0 for both AMI and control group, measured by the two different approaches, 2D and 3D were consistent, demonstrating insignificant inter- and intra-observer variability (ICC = 0.95, 95% CI: 0.96–0.99; ICC = 0.98, 95% CI: 0.91–0.99 for 2D and ICC = 0.96, 95% CI: 0.9–0.93; ICC = 0.94, 95% CI: 0.9–0.95 for 3D in AMI subjects). The mean LVEF by 2D and 3D was significantly lower in patients with AMI (41.2%±9.5% − 2D, 41.5%±8.5% − 3D) compared with controls (56%±1.9% − 2D, 55.4%±3.78 – 3D) (Table 1). At follow-up, in the AMI population there was a significant increase in LVEF at both T1 (45.8 ± 9.6% – 2D; 46.2 ± 8.4 – 3D) and T6 (49.16 ± 8.2% – 2D; 48.7 ± 7.3 – 3D).

Also, there was no correlation between different individual ILs and classical biological parameters, such as the number of leukocytes, the percentage of neutrophils, lymphocytes or erythrocytes sedimentation rate.

In the multiple regression equation of inflammation and Gensini score, only CRP and SDF-1α had statistical relevance (Table 3).

**Table 3.** The multiple regression equation for Gensini score in the acute myocardial group.

Independent variables	Standardized coefficient	Standard error	*r*_partial_	*t*	*p* Value
IL-1ß (pg/mL)	−0.151	0.013	−0.166	−5.60	.587
SDF-1α (pg/mL)	0.699	0.001	0.699	2.587	.036
CRP (mg/mL)	0.747	0.081	0.747	3.895	.02

CRP: C reactive protein; IL-1β: interleukin 1β; SDF-1α: stromal-derived factor 1α.

## Discussions

The results of our study pinpoint that the inflammatory process has important dynamics in the first month after AMI with marked changes in plasma concentrations, and normalization at 6 months after the acute ischaemic event. Interestingly, our study reveals that the level of inflammatory cytokines correlates with the severity of coronary lesions and the evolution of left ventricular ejection fraction at 1 month.

There is a statistically significant difference between CRP and SDF-1α values of patients with AMI and control group, and in the case of IL-1β it shows values at the limit of statistical significance. Our results sustain and enhance the inflammatory hypothesis of atherothrombosis, in line with trials such as CANTOS [13], COLCOT [22], LoDoCo 1 [23] and 2 [24]. All the mentioned trials show that the addition of anti-inflammatory drugs (Canakinumab or colchicine) reduces recurrent cardiovascular events compared with placebo generating the hypothesis that mitigating inflammation could supplementary reduce cardiovascular events independently of the secondary prevention therapies, including statins, blood pressure-lowering and antithrombotic therapies in acute, but also chronic coronary syndromes.

In subjects with AMI the mean values of CRP were higher compared to controls, while in the 6 months of follow-up, CRP showed constant decrease. It is to mention that the mean values of CRP were slightly higher in the control group when compared with other values mentioned in literature [13], due to the fact that the controls were not chosen from those without cardiovascular risk factors, but from those presenting with angina pectoris without significant coronary lesions. We found a positive correlation between CRP and the severity of coronary stenosis. The more elevated CRP values are at the moment of AMI, the more severe the coronary atherosclerotic lesions at angiography and the more reduced was LVEF at 1 month after AMI.

IL-1β showed lower concentrations in the subjects with AMI compared with a control group, while at 1 and 6 months after AMI the concentrations raised. The plasmatic increase of IL-1β’s concentration in the AMI group could be explained by the fact that at the time of the acute ischaemic event, IL-1β is the promoter of multiple cascade reactions, being considered an effector IL that disappears from circulation by specific receptor binding. Previous studies have found that IL-1β concentration is undetectable in healthy individuals and in patients undergoing AMI recovery [25]. Another explanation may be the lower detection limit of the kit used, which was approximately 1 pg/mL, which may decrease the IL’s detection sensitivity in patients with plasmatic values below this threshold. On the other hand, many IL-1β dosing difficulties are described in literature, many studies quitting the quantitation of this cytokine that binds very easily to various proteins and which can be altered and damaged during defrosting. Therefore, in some studies, it was preferred the analysis of the activity of IL-1β receptor which exhibits greater stability, but which does not describe as precise as IL-1β the functional activity of the molecule [26]. IL-1β at the time of AMI weakly correlated with the severity of coronary lesions (rho = 0.29), but strongly correlated with 2D LVEF at 1 month (rho = −0.8). Patients with lower levels of IL-1β at AMI, had higher LVEF at the one month and less severe coronary lesions.

SDF-1α had higher values in the group of patients with AMI, with permanent decrease during the 6-month follow-up, but never reaching the means of the control group. The values during hospitalization for AMI correlated with the severity of coronary lesions and with LVEF; higher the values of SDF-1α at T0, more severe the coronary involvement and more reduced the LVEF at 1 month. In the multiple regression equation of Gensini score, only CRP and SDF-1α predicted the severity of coronary lesions. SDF-1α is one major chemokine that stimulates progenitor cell mobilization from the bone marrow to the ischaemic tissues. Studies are inconsistent among results, some of them showing increased plasmatic values as we pinpointed in our study [18], in contrast with others revealing decreased levels [27]. These differences may be due to the various factors, such as the time of blood harvest (at three days in our trial compared with acute phase of MI in others) and the extend of myocardial infarction. Increased plasmatic levels are due to the up-regulation of SDF-1α expression after MI; accordingly, we found significant levels of SDF-1α compared with the control group. Previous studies evidence that SDF-1α is a prevalent rather local mediator than systemic for stem cell engraftment in the cardiac tissue [18]. Although there are other studies that show reduced plasmatic levels of SDF-1α in the acute settings of MI in relation to the increased binding of SDF-1 at its receptors CXCR4 and CXCR7 on the reactive blood cells [27], the precise plasmatic levels may depend upon situation on local concentration, duration, and time period after MI [28].

A larger study population is requested in order to correctly conclude about the potential role of inflammatory markers in the prediction of coronary lesions severity and LV dysfunction.

One drawback of this study is the low number of patients enrolled, therefore, we plan an increase of both control and AMI groups. Another bias could be represented by the effect of various drugs on the expression of ILs, for example, it is not known to what extent these observed differences cannot be due to therapy. Angiotensin-converting enzyme inhibitor or angiotensin receptor blocker therapy in AMI patients may result in a decrease of IL-1β concentration [29]. But, the major limitation of this study is the lack of histopathological evidence of coronary inflammatory process. Since no local IL-1β or SDF-1α concentrations were determined, no conclusion can be drawn regarding the potential role of CRP, IL-1β or SDF-1α in triggering inflammation at myocardial level. It can only be hypothesized according to the data obtained that the high plasmatic levels of CRP and SDF-1α, sign of a pro-inflammatory status are related with more severe coronary lesions and decreased LVEF.

All in all, even if the inflammatory hypothesis of atherosclerosis has more than five decades since first launched, it still has many facets to investigate, mainly the potential for inducing future innovative treatments.

## Data Availability

The data that support the findings of this study are available upon reasonable request.
